# N-Methyl-D-aspartate Glutamate Receptor Modulates Cardiovascular and Neuroendocrine Responses Evoked by Hemorrhagic Shock in Rats

**DOI:** 10.1155/2021/1156031

**Published:** 2021-08-13

**Authors:** Cristiane Busnardo, Aline Fassini, Bruno Rodrigues, José Antunes-Rodrigues, Carlos C. Crestani, Fernando M. A. Corrêa

**Affiliations:** ^1^Department of Drugs and Medicines, School of Pharmaceutical Sciences, São Paulo State University (UNESP), Araraquara, SP, Brazil; ^2^Department of Pharmacology, School of Medicine of Ribeirão Preto, University of São Paulo, Ribeirão Preto, SP, Brazil; ^3^Department of Adapted Physical Activity, Faculty of Physical Education, University of Campinas-UNICAMP, Campinas, SP, Brazil; ^4^Department of Physiology, School of Medicine of Ribeirão Preto, University of São Paulo, Ribeirão Preto, SP, Brazil

## Abstract

Here, we report the participation of N-methyl-D-aspartate (NMDA) glutamate receptor in the mediation of cardiovascular and circulating vasopressin responses evoked by a hemorrhagic stimulus. In addition, once NMDA receptor activation is a prominent mechanism involved in nitric oxide (NO) synthesis in the brain, we investigated whether control of hemorrhagic shock by NMDA glutamate receptor was followed by changes in NO synthesis in brain supramedullary structures involved in cardiovascular and neuroendocrine control. Thus, we observed that intraperitoneal administration of the selective NMDA glutamate receptor antagonist dizocilpine maleate (MK801, 0.3 mg/kg) delayed and reduced the magnitude of hemorrhage-induced hypotension. Besides, hemorrhage induced a tachycardia response in the posthemorrhage period (i.e., recovery period) in control animals, and systemic treatment with MK801 caused a bradycardia response during hemorrhagic shock. Hemorrhagic stimulus increased plasma vasopressin levels during the recovery period and NMDA receptor antagonism increased concentration of this hormone during both the hemorrhage and postbleeding periods in relation to control animals. Moreover, hemorrhagic shock caused a decrease in NOx levels in the paraventricular nucleus of the hypothalamus (PVN), amygdala, bed nucleus of the stria terminalis (BNST), and ventral periaqueductal gray matter (vPAG). Nevertheless, treatment with MK801 did not affect these effects. Taken together, these results indicate that the NMDA glutamate receptor is involved in the hemorrhagic shock by inhibiting circulating vasopressin release. Our data also suggest a role of the NMDA receptor in tachycardia, but not in the decreased NO synthesis in the brain evoked by hemorrhage.

## 1. Introduction

Hemorrhagic shock is a syndrome characterized by acute circulatory dysfunction due to a global poor blood flow distribution. Reduced tissue perfusion results in inadequate tissue oxygenation that, in turn, might evoke abnormal cell metabolism, cell death, and multiple organ failure [1–3]. The severity of the hemorrhagic shock state is directly related to the blood volume lost, in which losses greater than 30% of total blood volume are correlated to a high mortality rate [4–7]. It is worth mentioning that about 40% of trauma-associated deaths are due to uncontrolled bleeding and its consequences [8, 9], so clinical approach should be achieved as quickly as possible.

Under conditions of blood loss, a sequence of physiological events is triggered by baroreflex and atrial volume receptor activation. Initially, activation of sympathetic nervous system and the renin-angiotensin-aldosterone system maintains blood pressure despite a decrease in cardiac output [10–14], a period-denominated compensatory phase. Subsequently, if the hemorrhage is not reversed, the decompensatory phase is established when approximately 30% of total blood volume is lost. In the decompensatory phase, the intense vasoconstriction observed during the compensatory phase progresses to vasodilation due to activation of cardiac spinal signals [15, 16], which contribute to hypotension and catecholamine-resistant cardiovascular collapse [10, 17–20]. When bleeding is stopped, the recompensatory phase is initiated to increase circulating vasopressin, heart rate (HR), and cardiac sympathetic activity, which together mediates the mean arterial pressure (MAP) return to levels close to baseline values [21–23].

Several brain areas are involved in autonomic and neuroendocrine responses caused by bleeding [17, 24–29]. For instance, hemorrhage-associated cardiovascular and blood volume sensory signals from baroreflex and atrial volume receptors reach the central nervous system (CNS) in the nucleus tractus solitarius (NTS), which in turn sends this information to the brainstem and supramedullary structures [11, 30]. Although these pieces of evidence, the brain neurochemical mechanisms involved in hemorrhage-evoked physiological adjustments are still poorly known. In this sense, glutamate is the main excitatory neurotransmitter in the brain and is involved in cardiovascular and neuroendocrine control [31–35]. Nitric oxide (NO) is also a prominent signaling molecule in the CNS controlling physiological and pathological processes [36–39]. Although there is evidence of the presence of other nitric oxide synthase (NOS) enzyme isoforms in the brain [39, 40], the neuronal NOS (nNOS) seems to be the main isoform involved in NO synthesis in the CNS [41]. The nNOS is Ca^+2^-dependent, and activation of NMDA glutamate receptor has been described as a prominent mechanism involved in the activation of this NOS isoform in the brain [39, 40].

Electrophysiological and pharmacological studies have shown the participation of glutamatergic and nitrergic neurochemical mechanisms controlling cardiovascular function. For example, systemic vasopressin release is controlled by supramedullary structures, such as medial prefrontal cortex (mPFC), bed nucleus of the stria terminalis (BNST), paraventricular nucleus of the hypothalamus (PVN), periaqueductal gray matter (PAG), and supraoptic nucleus (SON) [32, 33, 42–44]. However, the role of the NMDA glutamate receptor in the control of cardiovascular and endocrine responses during hemorrhagic stimulus is poorly understood. Therefore, we hypothesized in the present study that glutamate acting via NMDA receptor is involved in hemorrhage-induced cardiovascular and endocrine responses. As stated above, activation of the NMDA glutamate receptor is a prominent mechanism involved in nNOS activation and NO release in the brain [39, 40]. However, the effect of hemorrhage in nitrergic signaling in supramedullary structures has never been reported. Therefore, we also evaluated whether changes followed control of hemorrhagic shock by the NMDA glutamate receptor in NO release in supramedullary structures.

## 2. Material and Methods

### 2.1. Animals

Experimental procedures were carried out following protocols approved by the Ethical Review Committee of the School of Medicine of Ribeirão Preto (protocol # 075/2015). Male Wistar rats weighing approximately 250 g were used in the present experiment. Animals were housed in plastic cages in a temperature-controlled room (25°C) in the Animal Care Unit of the Department of Pharmacology, School of Medicine of Ribeirão Preto. Animals were kept under a 12 : 12 h light-dark cycle (lights on between 06:00 and 18:00 h). Animals had free access to water and standard laboratory food, except during the experimental period.

### 2.2. Experimental Procedures

Rats were transported to the experiment room in their cages and were allowed at least 60 min to adapt to the experimental room conditions, such as sound and illumination, before starting the experiments.

#### 2.2.1. Effect of Systemic Treatment with MK801 on the Cardiovascular and Circulating Vasopressin Responses to Hemorrhage in Unanesthetized Rats

**Figure 1:**
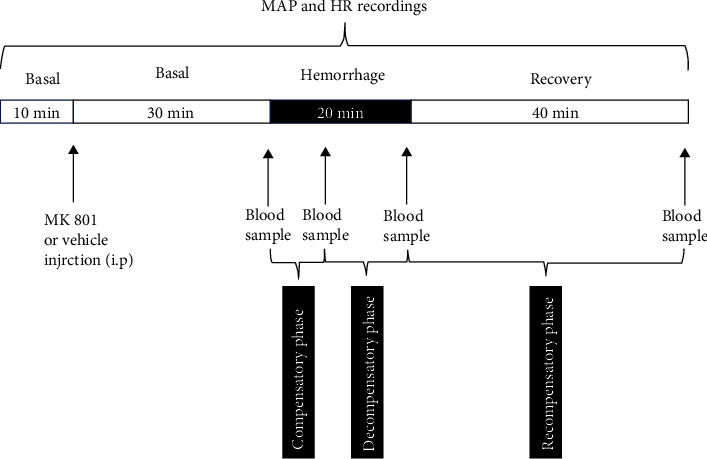
Schematic representation of the experimental protocol for evaluation of the effect of MK801 (selective NMDA glutamate receptor antagonist) on cardiovascular and circulating vasopressin changes evoked by hemorrhage. Please see the text for details.

An initial 10 min cardiovascular recording was performed to obtain basal blood pressure and heart rate (HR). Afterward, animals were treated intraperitoneally (i.p.) with either saline (vehicle, 1 mL/kg) or the selective noncompetitive NMDA glutamate receptor antagonist dizocilpine maleate (MK801, 0.3 mg/kg) 30 min before the hemorrhage onset [45]. The cardiovascular recording was continuously performed during the 30 min period after the treatment and the 20 min period of hemorrhage, ending 40 min after completing the hemorrhage. Blood samples for determination of plasma vasopressin concentration were collected immediately before the bleeding onset, at 7 and 20 min during the bleeding period and 40 min after the bleeding. The times for blood sampling were chosen because they represent the end of the compensatory, decompensatory, and recompensatory phases of the hemorrhagic shock in our experimental model [23].  Figure 1 presents the entire experimental protocol for evaluating cardiovascular and circulating vasopressin changes evoked by hemorrhage.

#### 2.2.2. Effect of Hemorrhage and/or Treatment with MK801 on NOx Levels in Supramedullary Structures

The quantification of nitrite (NO_2_) and nitrate (NO_3_) (NOx; spontaneous oxidation products of the NO) was utilized as an indirect measurement of NO production [45, 46]. For this, an independent set of animals about those used in the above protocol were divided into four groups: (i) vehicle-control, in which animals were treated with vehicle (saline, i.p., 1 mL/kg) and were not subjected to the hemorrhagic stimulus; (ii) vehicle-hemorrhage, in which animals were treated with vehicle (saline, i.p., 1 mL/kg) 30 min before the hemorrhage onset; (iii) MK801-control, in which animals were treated with the selective noncompetitive NMDA glutamate receptor antagonist MK801 (0.3 mg/kg) and were not subjected to the hemorrhagic stimulus; and (iv) MK801-hemorrhage, in which animals were treated with MK801 (0.3 mg/kg) 30 min before the hemorrhage onset [45]. Animals were decapitated immediately after bleeding, the brain was removed, and the structures of interest were dissected for NOx assay. For PVN and PAG, it was necessary to make a pool to reach sufficient samples so that the number of samples in the analysis of these structures is smaller compared to measurements in the amygdala, BNST, and mPFC.

### 2.3. Surgical Preparation

Animals were anesthetized with tribromoethanol (250 mg/kg, i.p.), and polyethylene catheters were implanted into the right femoral artery for cardiovascular recording and into the left femoral artery for blood withdrawal (hemorrhage). The catheters were exposed to the animals' dorsum and attached to the skin, allowing cardiovascular recording and blood withdrawal of unanesthetized rats in their cage [23]. Flunixin meglumine (2.5 mg/kg s.c.) was used for postoperation analgesia, and a poly-antibiotic solution containing streptomycin and penicillin (560 mg/mL/kg, i.m.) was administrated to prevent infection.

### 2.4. Cardiovascular Recording

For the cardiovascular recording, the catheter implanted into the femoral artery was connected to a pressure transducer, and the pulsatile arterial pressure (PAP) was recorded using an HP-7754A preamplifier (Hewlett Packard, Palo Alto, CA, USA) and an acquisition board (MP100A, Biopac Systems Inc., Goleta, Santa Barbara, CA, USA) connected to a personal computer. Mean arterial pressure (MAP) and heart rate (HR) values were derived from PAP recordings using the Acknowledge III software (Biopac Systems Inc., USA). The MAP was calculated according to the equation: diastolic pressure + (systolic‐diastolic)/3. The HR was calculated from PAP peak intervals integrated every 6 s [23].

### 2.5. Hemorrhage

All animals underwent a fixed volume hemorrhage of 24 mL/kg (estimated as 30% of total blood volume) throughout 20 min (1.2 mL/min/kg) [23]. This rate evokes clear periods of compensation, decompensation, and recompensation [21, 23]. Blood was withdrawn via the left femoral artery using a withdrawal pump (K.D. Scientific, Holliston, MA, USA) [23].

### 2.6. Plasma Vasopressin Measurement

Blood samples were collected in chilled plastic tubes containing heparin (10 *μ*L of heparin per mL of collected blood). Samples were centrifuged at 4°C, 3000 rpm for 20 min, and plasma was stored at −80°C until assay for determination of vasopressin levels. Plasma vasopressin levels were measured by specific radioimmunoassay after previous extraction from plasma using acetone and petroleum ether [47–49]. AVP assay detection limits ranged from 0.625 to 50 pg/mL, and intra-assay coefficient of variation was 16.8%. All samples from a single experiment were assayed in duplicate in the same assay.

### 2.7. Measurement of Nitrogen Oxides (NOx)

Immediately after the hemorrhage session, the rats were decapitated, and brains were removed. Prelimbic (PL) and infralimbic (IL) subregions of the mPFC, PVN, amygdala, BNST, dorsal PAG (dPAG), and ventral PAG (vPAG) were bilaterally dissected, homogenized in cold lysis buffer (20 mM Tris-HCl pH 7.6, 10% glycerol, 137 mM NaCl), and stored at -80°C. NOx concentrations were determined using a method adapted from P. N. Bories and C. Bories [50]. Briefly, before the NOx test, the homogenates were centrifuged at 20,000 g for 10 min at 4°C. After centrifugation, the supernatant was removed and incubated overnight with 0.5 mg/mL *β*-NADPH (Sigma-Aldrich, St. Louis, MO, USA) and 0.2 U/mL nitrate reductase (Sigma-Aldrich) in KH_2_PO_4_ buffer (0.2 M, pH 7.6) at 37°C for reduction of nitrate to nitrite. The nitrite level was determined by adding Griess reagent to (N-(1-naphthyl)ethylenediamine and sulfanilic acid dihydrochloride) (Molecular Probes, Eugene, OR, USA) samples, according to the manufacturer's instructions. After 10 min of incubation at room temperature, absorbance at 540 nm was determined, and nitrite concentrations were calculated from the standard sodium nitrite curve (Sigma-Aldrich, St. Louis, MO, USA). The protein content in the individual samples was measured using the Bradford reagent (Sigma-Aldrich, St. Louis, MO, USA); serum albumin was used as standard (BioRad, Wien, Austria). All measurements were performed in triplicate, and results were expressed in *μ*M NOx/*μ*g of protein [45, 51].

### 2.8. Drugs and Solutions

The selective noncompetitive NMDA glutamate receptor antagonist dizocilpine maleate (MK801) (Sigma-Aldrich, St. Louis, Missouri, USA), tribromoethanol (Sigma-Aldrich) and urethane (Sigma-Aldrich) were dissolved in saline (NaCl 0.9%). Flunixin meglumine (Banamine®, Schering Plough, RJ, Brazil) and the poly-antibiotic preparation of streptomycin and penicillin (Pentabiotico®, Fort Dodge, SP, Brazil) were used as provided.

### 2.9. Data Analysis

Data were expressed as the means ± SEM. Changes in MAP (*Δ*MAP) and HR (*Δ*HR) evoked by hemorrhagic stimulus were calculated concerning the mean values obtained during the 10 min before the bleeding onset. Data were initially subjected to D'Agostino-Pearson omnibus normality test, and analysis indicated a normal distribution for all results. Therefore, basal values of MAP and HR before and after pharmacological treatment were compared using Student's paired *t*-test. The time-course curves of *Δ*MAP, *Δ*HR, and AVP were analyzed using two-way ANOVA, with treatment (vehicle × MK801) as the main factor and time as a repeated measurement. The NOx levels were also analyzed using two-way ANOVA, with treatment (vehicle × MK801) and condition (control × hemorrhage) as main factors. When ANOVA indicated a significant interaction between the factors, the Bonferroni post hoc test was performed to identify specific differences. P < 0.05 was assumed as statistically significant.

## 3. Results

### 3.1. Effects of Systemic Treatment with MK801 on the Hemorrhage-Induced Cardiovascular Changes

Systemic (i.p.) treatment with saline (1 mL/kg, *n* = 6) did not affect baseline values of either MAP or HR (before: MAP = 109 ± 2.6, HR = 393 ± 7; after: MAP = 109 ± 2.4 mmHg, *t* = 0.67, *P* = 0.53; HR = 394 ± 5.8 bpm, *t* = 0.17, *P* = 0.88). Systemic treatment with the selective NMDA glutamate receptor antagonist MK801 (0.3 mg/kg, *n* = 7) also did not affect MAP or HR baseline values (before: MAP = 107 ± 3.8 mmHg, HR = 417 ± 16.6 bpm; after: MAP = 110 ± 2.5 mmHg, *t* = 1.6, *P* = 0.15; HR = 432 ± 5.3 bpm, *t* = 1.04, *P* = 0.34).

**Figure 2:**
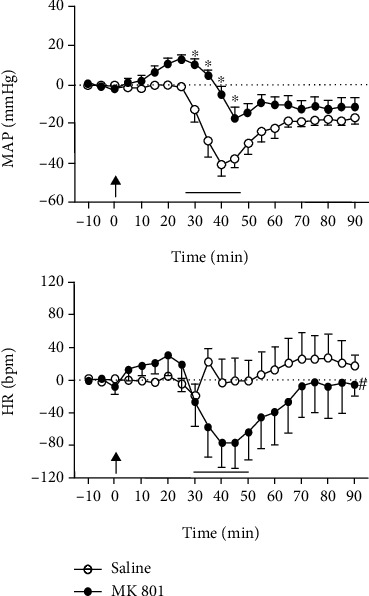
Time-course curves of the effect of systemic treatment with vehicle (saline, 1 mL/kg, *n* = 6) or the selective noncompetitive NMDA glutamate receptor antagonist MK801 (0.3 mg/kg, *n* = 7) in MAP and HR changes evoked by hemorrhage. Drug injections were made at time 0, as indicated by an arrow. The bleeding started at time 30 and finished at time 50; the black line represents the period of hemorrhage. Points represent the mean and bars the SEM. ^∗^Significantly different from control (vehicle) at the same time point (Bonferroni post hoc test, *P* < 0.05); # indicates main effect of treatment (two-way ANOVA, *P* < 0.05).

Two-way ANOVA of the time-course curves of *Δ*MAP indicated a significant influence of MK801 treatment [*F*_(1, 231)_ = 89.2, *P* < 0.0001] and an effect over time [*F*_(20, 231)_ = 14.42, *P* < 0.0001], the latter indicating a hemorrhage-induced hypotension. Besides, analysis indicated an interaction between treatment and time [*F*_(20, 231)_ = 3.63, *P* < 0.0001] (Figure 2). *Post hoc* analysis revealed that NMDA glutamate receptor blockade increased the latency to the onset of hypotension during hemorrhage and decreased the *Δ*MAP in relation to vehicle-treated animals (*P* < 0.05) (Figure 2).

Analysis of HR response revealed the effect of treatment with MK801 [*F*_(1, 231)_ = 8.82, *P* = 0.0033], but without the influence of time [*F*_(20, 231)_ = 0.91, *P* = 0.5751] and a treatment × time interaction [*F*_(20, 231)_ = 0.88, *P* = 0.6156]. *Post hoc* analysis revealed increased HR values during the posthemorrhage period in animals subjected to saline treatment (i.e., control) (saline basal: ΔHR = −0.57 ± 0.97; saline posthemorrhage period: ΔHR = 19.1 ± 2.52, *t* = 6.83, *P* = 0.0002) (Figure 2). Besides, a bradycardia during bleeding was identified in MK801-treated animals, so values of HR were lower in relation to control animals throughout the hemorrhage and posthemorrhage periods (*P* < 0.05) (Figure 2).

### 3.2. Effects of Systemic Treatment with MK801 on the Hemorrhage-Induced Increase in Circulating Vasopressin

**Figure 3:**
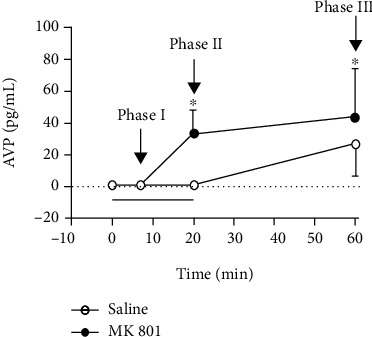
Time-course curves of the effect of systemic treatment with vehicle (saline, 1 mL/kg) or the selective noncompetitive NMDA glutamate receptor antagonist MK801 (0.3 mg/kg) in plasma vasopressin changes evoked by hemorrhage. The bleeding started at time 0 and finished at time 20; the black line represents the period of hemorrhage. Points represent the mean and bars the SEM. Saline = time 0: *n* = 16, time 7: *n* = 5, time 20: *n* = 6, time 60: *n* = 7; MK801 = time 0: *n* = 24, time 7: *n* = 10, time 20: *n* = 6, time 60: *n* = 7. ^∗^Significantly different from control (vehicle) at the same time point (Bonferroni *post hoc* test, *P* < 0.05).

Systemic administration of MK801 did not affect the basal values of plasma vasopressin when compared to saline-treated animals (saline = 1 ± 0.07 pg/mL, *n* = 12; MK801 = 1.2 ± 0.2 pg/mL, *n* = 12; *t* = 0.9, *P* = 0.3872). Two-way ANOVA indicated a significant effect of MK801 on hemorrhage-evoked increase in plasma vasopressin concentrations [*F*_(1, 73)_ = 19.4, *P* < 0.0001]; a significant effect over time [*F*_(3, 73)_ = 36.6, *P* < 0.0001], indicating an increase in vasopressin levels; and an interaction between treatment and time [*F*_(3, 73)_ = 7.5, *P* = 0.0002] (Figure 3). *Post hoc* analysis revealed that hemorrhage caused an increase in AVP plasma levels at posthemorrhage period in vehicle-treated animals when compared to baseline values (saline basal: AVP = 1 ± 0.07 pg/mL; saline phase III: AVP = 26.98 ± 7.83 pg/mL) (Figure 3). Besides, MK801 increased plasma vasopressin concentration in relation to the vehicle group at 20 min of hemorrhage and 40 min after ending the bleeding (Figure 3).

### 3.3. Effects of Systemic Treatment with MK801 and/or Hemorrhage on NOx Levels in the Brain

**Figure 4:**
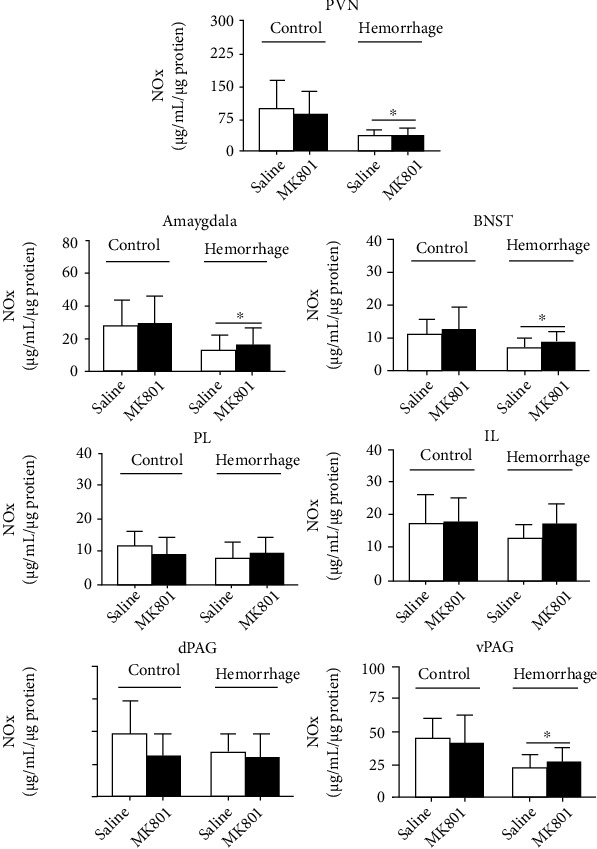
Effect of hemorrhagic shock on NOx levels in the paraventricular nucleus of hypothalamus (PVN) (control-saline: *n* = 6, control-MK801: *n* = 9; hemorrhage-saline: *n* = 6, hemorrhage-MK801: *n* = 6), amygdala (control-saline: *n* = 15, control-MK801: *n* = 24; hemorrhage-saline: *n* = 18, hemorrhage-MK801: *n* = 20), bed nucleus of the stria terminalis (BNST) (control-saline: *n* = 18, control-MK801: *n* = 25; hemorrhage-saline: *n* = 17, hemorrhage-MK801: *n* = 21), prelimbic cortex (PL) (control-saline: *n* = 17, control-MK801: *n* = 27; hemorrhage-saline: *n* = 18, hemorrhage-MK801: *n* = 21), infralimbic cortex (IL) (control-saline: *n* = 17, control-MK801: *n* = 25; hemorrhage-saline: *n* = 17, hemorrhage-MK801: *n* = 22), dorsal periaqueductal gray matter (dPAG) (control-saline: *n* = 6, control-MK801: *n* = 6; hemorrhage-saline: *n* = 8, hemorrhage-MK801: *n* = 8), and ventral periaqueductal gray matter (vPAG) (control-saline: *n* = 7, control-MK801: *n* = 7; hemorrhage-saline: *n* = 6, hemorrhage-MK801: *n* = 6) of animals treated intraperitoneally with vehicle (saline, 1 mL/kg) or the selective noncompetitive NMDA glutamate receptor antagonist MK-801 (0.3 mg/kg). The columns represent mean and bars the SEM. ∗ indicates main effect of treatment (two-way ANOVA, *P* < 0.05).

Hemorrhagic shock caused a significant reduction in NOx levels within the PVN) [*F*_(1, 23)_ = 9.69, *P* = 0.0049], amygdala [*F*_(1, 74)_ = 20.21, *P* < 0.0001], BNST [*F*_(1, 77)_ = 9.52, *P* = 0.003], and ventral PAG (vPAG) [*F*_(1, 22)_ = 9.68, *P* = 0.005] (Figure 4). However, analysis did not indicate either effect of MK801 treatment [PVN: *F*_(1, 23)_ = 0.13, *P* = 0.73; amygdala: *F*_(1, 74)_ = 0.74, *P* > 0.05; BNST: *F*_(1, 77)_ = 2.55, *P* > 0.05; vPAG: *F*_(1, 22)_ = 0.042, *P* > 0.05] or interaction between treatment and condition [PVN: *F*_(1, 18)_ = 0.001, *P* > 0.05; amygdala: *F*_(1, 74)_ = 0.1, *P* > 0.05; BNST: *F*_(1, 77)_ = 0.005, *P* > 0.05; vPAG: *F*_(1, 22)_ = 0.42, *P* > 0.05] (Figure 4).

Hemorrhagic shock did not change NOx levels within the prelimbic (PL) [*F*_(1, 79)_ = 2.73, *P* > 0.05] and inframbic (IL) [*F*_(1, 77)_ = 2.1, *P* > 0.05] subregions of the mPFC, as well as in the dorsal PAG (dPAG) [*F*_(1, 24)_ = 1.2, *P* > 0.05] (Figure 4). Analysis also did not indicate the effect of MK801 treatment [PL: *F*_(1, 79)_ = 0.053, *P* > 0.05; IL: *F*_(1, 77)_ = 2.53, *P* > 0.05; dPAG: *F*_(1, 24)_ = 2.46, *P* > 0.05] or interaction between treatment and condition [PL: *F*_(1, 79)_ = 2.87, *P* > 0.05; IL: *F*_(1, 77)_ = 1.51, *P* > 0.05; dPAG: *F*_(1, 24)_ = 0.72, *P* > 0.05] (Figure 4).

## 4. Discussion

We identified in the present study that systemic treatment with the selective NMDA glutamate receptor antagonist MK801 delayed and decreased the magnitude of the hypotension evoked by hemorrhage. Moreover, treatment with MK801 caused a bradycardia during hemorrhage, so HR values were lower throughout the bleeding and posthemorrhage periods in relation to saline-treated animals. Moreover, NMDA receptor antagonism facilitated the circulating vasopressin increase. We also observed that hemorrhagic stimulus decreased NOx concentration in the PVN, BNST, amygdala, and vPAG, but without affecting the levels in the mPFC (i.e., PL and IL) and dPAG. Systemic treatment with MK801 did not affect NOx levels in any structure evaluated.

### 4.1. Role of NMDA Glutamate Receptor in Hemorrhagic Shock

Previous studies have demonstrated that the tachycardiac response evoked by hemorrhage is mediated by sympathetic mechanisms [10, 23, 52]. Therefore, hemorrhage-caused bradycardia response in animals systemically treated with MK801 provides evidence that activation of the NMDA glutamate receptor mediates the sympathetically mediated HR increase. The bradycardia observed in MK801-treated rats is possibly related to an increase in parasympathetic tonus to the heart. In fact, previous studies identified that hemorrhage causes coactivation of cardiac sympathetic and parasympathetic systems in rats [53, 54]. Accordingly, we identified previously that local NMDA receptor blockade in the PVN shifted the tachycardia evoked by hemorrhage into bradycardia in rats [23].

Treatment with MK801 also delayed and reduced the magnitude of the hemorrhage-evoked hypotension, thus suggesting that activation of NMDA receptors contributes to hemorrhagic shock. The reduced hypotension in MK801-treated rats was followed by a facilitation of vasopressin release into the bloodstream. It is worth mentioning that such as observed in the present study in vehicle-treated animals, previous studies reported that an increase in plasma vasopressin concentrations is observed mainly in the posthemorrhage period, so this endocrine response is a prominent mechanism for the recovery of MAP during the recompensatory phase [21, 22]. Therefore, results presented here indicate that NMDA receptor activation inhibits vasopressin release during both decompensatory and recompensatory phases of the hemorrhagic shock.

Vasopressin is released into the bloodstream from magnocellular neurosecretory neurons located in the PVN and SON, regulated mainly by glutamatergic neurotransmission [34, 35]. In this sense, stimulation of vasopressin release from PVN and SON magnocellular neurons by glutamate has been demonstrated to be mediated mainly by non-NMDA receptors [47, 55, 56]. The idea that a non-NMDA glutamate receptor rather than an NMDA receptor is involved in vasopressin release is supported by previous evidence that vasopressin-mediated responses elicited by activation of other brain structures (e.g., PAG, BNST, and lateral septal area) were inhibited in animals pretreated with non-NMDA, but not NMDA, glutamate receptor antagonist in the PVN [57–59]. Conversely, NMDA receptor stimulation inhibits local magnocellular neurons, reducing vasopressin secretion into the systemic circulation [47]. Accordingly, intra-PVN microinjection of a selective NMDA glutamate receptor reduced the hemorrhage-induced hypotension through peripheral vasopressinergic mechanisms [23]. Taken together, these pieces of evidence indicate the PVN as a potential site whereby MK801 acted to inhibit hemorrhagic shock through the facilitation of vasopressin release. In this sense, inhibition of vasopressin release in the PVN by NMDA receptor might be mediated by activation of local GABAergic interneurons, which in turn inhibits the AVP magnocellular neurons. Accordingly, GABAergic neurotransmission activation inhibits the firing activity of neurosecretory neurons in the hypothalamus [60, 61].

Although the evidence presented above strongly indicates the PVN as a site involved in the effect of MK801 reported in the present study, previous studies have demonstrated that systemic administration of MK801 can act in several other brainstem and forebrain structures controlling vasopressin release [45, 62, 63]. Therefore, the involvement of NMDA receptors in hemorrhagic shock is potentially a general brain mechanism mediated by other regions in addition to the PVN. Thus, the results obtained in the present study provide initial evidence of the blockade of NMDA receptor as a potential new therapeutical target for the treatment of hemorrhagic shock. In this sense, it is worth mentioning that several NMDA receptor antagonists are being tested in clinical trials to treat psychiatric disorders, and some are already available clinically [64–66]. Therefore, the hemorrhagic shock might be an additional disorder in which these drugs might be an effective treatment.

### 4.2. Effect of Hemorrhage and NMDA Receptor Blockade on NOx Levels in the Brain

As stated above, activation of the NMDA glutamate receptor is a prominent mechanism involved in nNOS activation and NO release in the brain [39, 40]. Besides, brain nitrergic signaling has been implicated in regulating cardiovascular function and circulating vasopressin levels [36–38]. Therefore, we evaluated the effect of hemorrhage and systemic treatment with MK801 in NOx levels in supramedullary brain structures related to cardiovascular and circulating vasopressin control. Our data showed that hemorrhagic shock caused a drop in NOx levels in the PVN, amygdala, BNST, and vPAG, indicating a decreased NO synthesis. Regarding the vasopressin release, all these supramedullary structures are involved in controlling vasopressin release into the bloodstream [47, 67–69]. Although the specific role of local nitrergic neurotransmission in the control of vasopressin release in most of these structures has not been directly assessed, studies have indicated that centrally produced NO inhibits vasopressin release [38]. For instance, intracerebroventricular administration of a nonselective NOS inhibitor increased circulating vasopressin concentration [70]. Therefore, decreased local NO release in the PVN, amygdala, BNST, and vPAG is possibly related to hemorrhage-evoked vasopressin release.

This control seems to be site-specific regarding a possible role of the changes in NOx levels in autonomic and cardiovascular responses to hemorrhage. Concerning the PVN, in addition to magnocellular neurosecretory neurons, this hypothalamic nucleus is also composed of parvocellular neurons that modulate the sympathetic nervous system [71]. In this sense, PVN neurons projecting to medullary autonomic centers express nNOS [72]. However, previous studies provided paradoxical evidence regarding the role of PVN nitrergic neurotransmission in regulating autonomic activity and cardiovascular function, and the discrepancy seems to be related to anesthesia [73]. However, increases in blood pressure and HR were reported following PVN pretreatment with nonselective NOS inhibitors in anesthetized and unanesthetized rats [74–76], supporting the idea that NO in the PVN tonically suppresses sympathetic activity [72]. Therefore, the decrease in NO release in the PVN during hemorrhage might be involved in sympathetic activation observed during the compensatory and recompensatory phases of the hemorrhagic shock.

The ventrolateral PAG (vlPAG) plays a crucial role in the hypotensive response to blood loss by inhibiting sympathetic activity, most likely by activation of vasodepressor signals in the caudal midline medulla (CMM) [21, 77–79]. The role of local nitrergic neurotransmission in this sympathoinhibitory response has never been evaluated. However, previous studies reported that the NO precursor L-arginine microinjection into the PAG reduced the pressor response evoked by static muscle contraction [43]. Besides, microinjection of a nonselective NOS inhibitor in the vPAG inhibited the depressor response to acupuncture [44]. These results indicate that NO seems to activate sympathoinhibitory neurons present in the vPAG. Therefore, the decrease in NOx levels in the vPAG might be involved in sympathetic activation-evoked hemorrhage.

Previous studies also indicated a prominent role of BNST nitrergic neurotransmission in autonomic and cardiovascular control. For instance, blockade of nNOS in the BNST enhanced baroreflex-mediated bradycardia and HR increase to emotional stress [80, 81]. Although this evidence indicates a modulation toward HR reduction, the NO seems to have a dual role in the BNST, including tachycardiac and bradycardiac responses [82]. Therefore, the reduction in NOx levels in the BNST might involve cardiovascular and autonomic changes in all phases of hemorrhagic shock. However, to the best of our knowledge, the role of the BNST in responses evoked by hemorrhage has never been documented. Regarding the amygdala, although there is evidence that NO donor causes activation of neurons in the central nucleus of the amygdala (CeA) [83], a role of local nitrergic neurotransmission in control of cardiovascular function in the amygdaloid nuclei has never been documented.

Unexpectedly, systemic treatment with MK801 did not affect NOx levels under either resting or hemorrhage conditions in any structure evaluated. This finding is not due to the ineffectiveness of the treatment, once we have previously reported that this same pharmacological treatment inhibited an increase in brain NOx levels evoked by treadmill exercise in rats [45]. Therefore, under the experimental conditions of the present study, NO seems to be released in the brain by mechanisms independent of the NMDA receptor. Accordingly, *in vitro* studies provided evidence that activation of muscarinic cholinergic receptors induced synthesis of both NO and cyclic guanosine cyclic monophosphate (cGMP) [84, 85], and the muscarinic receptor/nNOS/cGMP pathway seems to be involved in cardiovascular regulation in the brain [86]. Furthermore, an increase in the nNOS enzymatic activity might occur through its binding to serotonin transporters in the plasma membrane [87, 88]. Another possibility for the absence of effect of MK801 in NOx levels is that NO is released by isoform other than nNOS, which are not related to NMDA receptors. For instance, NO release found eNOS present in blood vessels which might signal to neurons [89].

In summary, our results indicate that activation of NMDA glutamate receptor is involved in hemorrhage-induced hypotension and tachycardia. The involvement in hypotension seems to be mediated by an inhibition of the vasopressin release into the circulation during the decompensatory and recompensatory phases of the hemorrhagic shock. Data reported in the present study also evidence that hemorrhage decreases the NO production in the PVN, amygdala, BNST, and vPAG (but not in the mPFC and dPAG) through a mechanism independent of NMDA glutamate receptor.

## Data Availability

Data available is on request.
